# The role of lifestyle in perpetuating substance use disorder: the Lifestyle Balance Model

**DOI:** 10.1186/1747-597X-10-2

**Published:** 2015-01-17

**Authors:** Glyn Davies, Sarah Elison, Jonathan Ward, Alexandre Laudet

**Affiliations:** Breaking Free Group, 274 Deansgate, Manchester, M3 4JB UK; Centre for the Study of Addictions and Recovery, National Development and Research Institutes, 71 West 23rd Street,4th Floor, New York, NY 10010 USA

## Abstract

Conceptualizing aetiology underpinning an individual’s substance use disorder (SUD) not only facilitates insight and understanding, but also serves to identify targets for treatment and aid practitioners in selecting the most appropriate interventions. There is now a wealth of literature on aetiology and treatment approaches, and in more recent years, also literature to support the concept of ‘recovery’ from a condition which was previously thought of as a chronic, relapsing condition. The burgeoning literature around research into recovery is revealing how recovery can best be defined and what factors might be associated with recovery from SUD. To add further to this growing body of literature, a new six-domain, explanatory biopsychosocial model of substance dependence and recovery, the Lifestyle Balance Model (LBM) is proposed. Based on research findings and theory reported in the literature, the LBM is a generic model depicting six domains of biopsychosocial functioning and includes within it the role of lifestyle. The LBM has been constructed as a domain model, allowing conceptualisation of the relationships between the six domain areas that perpetuate dependence and may also be associated with recovery from SUD, providing service users and clinicians with a tool for the delivery of case formulation and identification of target areas for intervention.

## Introduction

Understanding the aetiology of substance use is important as this may facilitate identification of potential areas of difficulty in functioning which may thereby serve as target areas for appropriate treatment and intervention [1]. However, for even the most experienced clinicians, it can sometimes be difficult to identify and conceptualize the many contributory factors that might underpin an individual’s substance use and how these factors may be inter-related. Further complicating matters is that there may be important, sometimes causal relationships between each of the contributory factors underpinning substance use, including mental health and social functioning difficulties, relationship problems and other stressful life circumstances [2]. There are also multiple ways in which substance use is referred to in the literature, with a number of distinct diagnostic categories describing different kinds of substance use and differing degrees of severity of dependence [3], leading some researchers to refer to the broader, general category of ‘substance use disorders’ (SUD) (e.g.[4]). For brevity throughout this article, SUD will be used to refer to the broad range of forms of substance use.

Exploration and understanding of the causal relationships between each of these areas of difficulty forms an important first step in the treatment process [5]. This may be particularly important for individuals who are ‘dually diagnosed’, i.e. are experiencing comorbid mental health difficulties such as depression and anxiety alongside their SUD, as often mental health difficulties may be causally related to SUD [6]. The literature demonstrates that many individuals, as much as 75 – 85% of the substance using population, may self-medicate with substances in an attempt to alleviate mental health difficulties [7], although for many their use of substances may exacerbate the severity of their mental health difficulties [1, 7]. This would suggest that the majority of individuals with SUD could be deemed as being dually diagnosed and that comorbid mental health issues should be considered alongside the other aetiological biopsychosocial factors associated with substance use.

Biopsychosocial domain models may be useful in facilitating the understanding of aetiology as they allow multiple aspects of, and influences on, human functioning to be understood using a visual format [8, 9]. Most often domain models comprise a series of ‘nodes’ representing each relevant aspect of functioning and influence, with lines and arrows of direction between nodes demonstrating the relationships between them. Domain models that facilitate conceptualisation of aetiology of SUD, and identification of potential intervention targets, have become more common in recent years [8, 9], providing a staple approach in the addiction field in the UK, the US and elsewhere [10]. The utility of domain models allows service users and practitioners to make sense of the various contributing factors that perpetuate the cycle of SUD, and helps identify areas where change might be needed.

There are several benefits to using domain models, including their simplicity and clarity in conveying relationships of cause and effect, and their visually impactful format, which can help addicted individuals to remember and recall the information contained within them. Due to their emerging evidence base and ease of implementation across the UK treatment sector, domain models have been recommended by Public Health England (PHE), the Welsh Assembly and Scottish Governments as a method for broadening the clinical application of psychosocial interventions across SUD treatment. This far reaching initiative was introduced through the International Treatment Effectiveness Project [ITEP: 5], resulting in the utility of mapping techniques becoming the norm within UK clinical practice.

Domain models have been developed that are appropriate for use during specific stages of the treatment process, including treatment engagement [10], relapse prevention [11, 12], and mutual-aid approaches such as SMART recovery [13, 14]. However, as yet, there is no single model that can be used throughout treatment regardless of the treatment stage, whether it be initial engagement with the process or the point at which abstinence has been achieved or sustained. Nor does any domain model exist that takes into account the role of *lifestyle* in the aetiology and maintenance of SUD. Lifestyle, whilst often overlooked in much SUD research, has been emerging in the literature over the past decade as an area for consideration in aetiology and treatment (e.g. [15–17]).

Although there is no consensus within the academic literature about how ‘lifestyle’ is defined, the authors intend the term to mean *the key ingredients that make up a person’s health and wellbeing, including (but not limited to) relationships, employment status and accommodation*. The impact that lifestyle *balance* can have on SUD cannot be overstated. For example, aspects of lifestyle imbalance, such as unemployment, relationship breakdowns and homelessness, are likely to increase the risk of and exposure to substance use as a coping mechanism. By contrast, aspects of lifestyle balance, such as stable employment, relationships and accommodation, are likely to strengthen a person’s resilience to prevent or overcome substance use difficulties.

**Figure 1:**
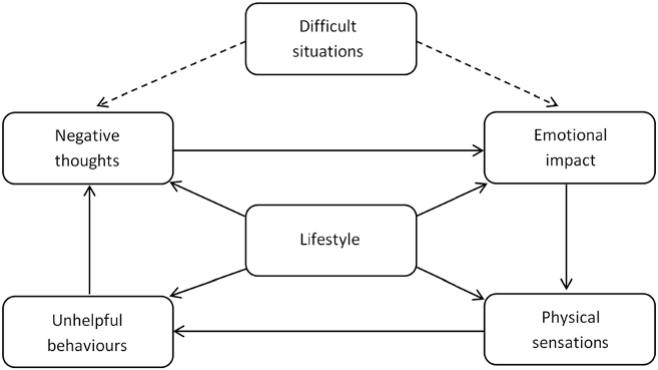
**The Lifestyle Balance Model.**

This paper therefore posits a rationale for and describes a new conceptual model of SUD that incorporates the role of lifestyle balance, the Lifestyle Balance Model (LBM- see Figure 1). The LBM provides service users and clinicians with a domain model to aid the understanding of the aetiology and conceptualisation of SUD. The LBM also provides stakeholders with a domain model that can be applied at all stages of the treatment journey, not only to help them understand SUD but also to provide a tool for targeted treatment intervention.

### The use of domain models in the treatment of SUD

The components of domain models may be useful not only in conceptualizing the aetiology of SUD and identifying target areas for change, but also for highlighting potential domains of biopsychosocial functioning that may influence recovery and risk of relapse, such as those included in the cognitive behavioural therapy (CBT) domain models commonly used in mental health [18, 19]. Domain models take into account the causal roles of cognitions, emotions, behaviours, physiology and social context in the aetiology of an individual’s mental health difficulties. Taking this approach may also be useful to clinicians working with individuals with SUD when developing treatment plans, and to addicted persons themselves, as it helps them to understand the inter-relationships among complex factors that may be contributing to their SUD.

In the UK, biopsychosocial models based on those used in the mental health field [2, 13, 20–22] are influencing the wider dissemination and implementation of psychosocial interventions in the SUD treatment sector. One example is the roll out of the International Treatment Effectiveness Project (ITEP: 5, [23, 24]), with organisations and clinicians being encouraged to adopt domain models (see http://www.nta.nhs.uk/uploads/nta_itep_implementing_psychosocial_interventions_for_adult_drug_misusers_rb34.pdf). Within the UK SUD sector, the use of domain models is more commonly referred to as ‘node-link mapping’ [8, 9], a technique that allows an individual to visualise and make sense of multiple parts of their life and how they each impact one another.

There has been debate among scientists and academics around the nature of the factors underpinning SUD and how these might interact, and so theories about the causes of SUD and the factors that maintain it are wide-ranging. Theories include the disease model (e.g. [25, 26]), genetic (e.g. [27]), neurobiological (e.g. [28, 29]), social-cognitive (e.g. [30]), social-learning (e.g. [31]), behavioral (e.g. [32]), cultural (e.g. [33]) and psychological perspectives (e.g. [20]).

Each discipline-specific theory may have a contribution to offer in terms of how it conceptualizes the aetiology of SUD and the factors that maintain it, and, conversely, what factors may also be associated with its remission. However, making these highly focused theoretical approaches easy to understand in the clinical setting can be challenging, limiting their intended usefulness. A broader, more holistic and generic approach to domain models has the potential to provide the basis for ‘clinical’ or ‘case’ formulation [34, 35], and can be personalised to individual service users. One model that works particularly well in translating theory into clinical practice is a cognitive-behavioral model known as the ‘five factor model’ proposed by Greenberger and Padesky [18]. The model explains the inter-relationships between five domains of biopsychosocial functioning; cognitions, emotions, biological/physiological, behaviors and environment, and how they are associated with an individual’s mental health.

### Factors maintaining and exacerbating SUD: the role of ‘lifestyle’

The lifestyle many individuals with SUD lead can be described as chaotic, characterised by unstable housing, unemployment, financial difficulties, committing crimes and social relationships centred around substance use [36]. Leading such a lifestyle may result in negative consequences that may serve to further maintain and exacerbate substance use [36]. These negative consequences include children being taken away from drug using parents by child care authorities such as Child Protective Services in the United States or Social Services in the UK [37], the loss of employment [38] or housing opportunities [39], financial insecurity [36], offending behavior [40] and mental and physical health problems [41]. All of these consequences are stressful and therefore may sustain SUD as substances are often used as a coping mechanism, despite their deleterious effect on one’s abilities to fulfil personal responsibilities [42].

The social networks an individual belongs to also play a key role in determining the kind of lifestyle they lead and the impact of this lifestyle on their health [43, 44], and so the extent of substance use occurring in these social networks may also influence an individual’s own level of use [45, 46]. As the use of substances becomes an integral part of daily life, the individual is likely to gravitate towards others with similar patterns [47], enhancing both integration in the substance using culture and exposure to substances. That further reinforces the negative impact of these social networks through the choices individuals make, so potentially contributing to life becoming more chaotic and unmanageable, resulting in further use of substances as they further engage in this behaviour as a means of attempting to cope with the stresses of life. Social epidemiological research also suggests that having a lifestyle characterised by the inclusion of substance users within one’s social networks may increase the likelihood of an individual also using substances [48, 49]. This may be due in part to the effect environmental stimuli related to substance use may have in eliciting cravings [50], social learning and reinforcement effects [51] and the ‘normalisation’ of SUD behavior within substance using social networks [52].

The importance of lifestyle in influencing health behaviors has been a focus for discussion in the literature around general health (e.g. [53]) and was first discussed in relation to SUD and relapse by Alan Marlatt and colleagues [54]. The authors define ‘lifestyle balance’ as: *“…the degree of equilibrium that exists in one’s daily life between the variety of activities a person engages in and the effects of those activities on one’s level of health and wellbeing (…) lifestyle balance refers to the amount of stress in a person’s daily life compared with stress reducing activities [and]…is also related to diet, social relationships, and spiritual endeavours”* ([54] pg 38). In Marlatt’s writing, lifestyle is conceptualized as important in SUD because the degree of lifestyle balance or imbalance might influence one’s desire for indulgence and gratification from substances. An imbalanced, chaotic lifestyle might be experienced as stressful and therefore may be more likely to lead to the use of psychoactive substances, either for pleasure or for stress alleviation.

It should be noted that despite the potential importance of lifestyle factors to SUD, the concept (and the term) should be used carefully in this context. In modern parlance the term ‘lifestyle’ often connotes a degree of *choice* on the part of the individual, especially given the current focus on encouraging the general population to engage in behaviors that characterise a healthy ‘lifestyle’ [55]. Lifestyle is often portrayed by the media as characterising the ways in which an individual *chooses* to live. Thus the use of the term in the context of *addiction* may elicit concerns if it is interpreted as suggesting that the use of substances among dependent persons is a lifestyle ‘choice’. Indeed, a lack of control over one’s substance use is one of the diagnostic criteria for SUD within the DSM-V [56], clearly implying an absence of choice. The term ‘lifestyle’ is therefore used here not to imply choice but instead to refer to the life context of the individual with SUD.

### Recovery from SUD and the role of lifestyle

In addition to playing a role in influencing SUD, lifestyle may play a role in shaping ‘recovery’. However, just as there are many ways of interpreting and defining lifestyle, there are also many definitions of recovery, and recent attempts to define the concept have resulted in some variation [57–60]. However, despite this variability, there is some consensus that recovery is a process whereby individuals improve various aspects of their health, wellbeing and functioning to reach their full potential alongside reduced consumption of, or complete abstinence from, substances [61]. Recovery-oriented interventions can have a positive impact on an individual’s lifestyle, thoughts, feelings and behaviors, and often aim to strengthen resilience and build ‘recovery capital’ [62, 63], the terms used to describe resources an individual has at their disposal to facilitate their recovery. These resources may be internal and include self-efficacy and motivation, or external and include social support networks and a stable and safe place to live, amongst other things [62, 63].

As previously described, ‘recovery’ is best conceptualized as a broad, multidimensional construct that goes beyond simply abstaining from the consumption of substances [64–67]. Rather, it is a process of rehabilitation that requires self-directed change and life transformation [68, 69]. Indeed, some have suggested that *habilitation* may be a more appropriate description of the process than *rehabilitation*, which often implies rebuilding a life that was once lived and then lost. However, this process is more about the creation of for the first time, of a healthy, functional and balanced lifestyle [70, 71].

The importance of lifestyle factors to promoting recovery is supported by the literature. In particular, research has documented the value of recovery-focused social support networks [72], having a stable place of residence [73], and being engaged in education, training or employment [74], all of which are key aspects of lifestyle. Making improvements in interpersonal relationships, financial and housing arrangements, health behaviors, education and employment, are cited as self-reported priorities among individuals in recovery [69]. A recent national study of over 3,000 persons in recovery in the US has documented significant improvements in these domains as a function of recovery, relative to when individuals were actively using substances [75]. Moreover, the amount of improvement increased gradually as recovery duration increased. In another study of formerly drug dependent persons, overall quality of satisfaction with a number of aspects of life including, social support, housing and employment, increased significantly relative to levels observed in active addiction, as recovery progressed; while stress, a predictor of relapse, decreased [76].

The need to enhance positive and balanced lifestyle factors to promote and support recovery from SUD is reflected in the national drug policy of several countries. In the United States, the national drug control strategy [77, 78] includes the goal of expanding support for recovery through community-based programs such as Recovery Oriented Systems of Care [79], collegiate recovery programs (ROSC: [75]), and recovery high schools [75]. The focus on advancing recovery by increasing an individual’s recovery capital through the promotion of health and lifestyle balance is also evident in UK policy, where Public Health England highlights the importance of three lifestyle factors, ‘jobs, homes and friends’, to building recovery capital and achieving recovery [80]. Indeed, one could argue that the concepts of ‘lifestyle’ and ‘recovery capital’ share much in common, as recovery capital describes functioning in many areas of an individual’s life that could be conceptualised as their lifestyle.

‘Recovery capital’ is a concept that tends to be understood by two groups, those operating within the SUD sector and more broadly the ‘recovery community’, i.e. those in recovery from SUD. Therefore, very few people outside of the SUD sector and recovery community would recognise or understand the concept of recovery capital. Given the degree of overlap between the concepts of recovery capital and lifestyle, using the term lifestyle instead may make allow a broader audience to understand the processes involved in recovery from SUD.

Although further exploration of the commonalities and differences between the two concepts would be informative, such analysis lies outside of the scope of this paper. However, the inclusion of lifestyle factors in an explanatory framework of SUD has the potential not only to highlight important contextual factors that perpetuate the cycle of SUD, but also to draw the focus of clinicians to a domain that, if targeted with effective interventions, may help build recovery capital and foster progress towards recovery.

### The Lifestyle Balance Model (LBM)

The above discussion highlights the potential importance of including lifestyle factors within biopsychosocial domain models for conceptualizing SUD, and the relevance of lifestyle factors to building recovery capital and achieving recovery. A new explanatory domain model for understanding SUD and recovery, the Lifestyle Balance Model (LBM), is therefore proposed as a means to explicate an individual’s circumstances, and enable areas of concern to be identified and appropriate interventions to be targeted. The LBM is an extension of the five-factor cognitive-behavioral model comprising cognitions, emotions, physiological factors, behavior and environment, commonly used within the mental health sector, and adapted by the authors to explain the specific experience of SUD. In the LBM, a sixth domain, ‘lifestyle’, is added to take into account drivers of SUD that are not included in the five-factor model. Thus, the lifestyle domain of the model incorporates general health and wellbeing, interpersonal relationships and social networks, material resources (such as finances), daily occupation (work, education or training), and housing/accommodation.

The LBM is based on standard CBT case formulation models [18, 19]. It allows conceptualisation of the impact that difficulties in six domains of biopsychosocial functioning may have on the individual. Using the LBM (represented graphically in Figure 1) enables both service users and clinicians to formulate a CBT-based, personalised model of an individual’s circumstances and how these domain areas may be contributing to their SUD, and by extension to their recovery.

Although the domains of functioning included in the LBM are generic and could be used to explain a range of human behavioral repertoires, such as health behaviors like physical activity and diet, in this instance, the relevance of the LBM domains to substance use is of central interest. The domains of the LBM and how they may be relevant to the behavioral repertoires characteristic of SUD are as follows.

‘Difficult situations’ are troubling situations in a person’s life or environment that they may be having difficulties coping with, such as exposure to substances or conflict with a partner. These difficult situation may result in ‘negative thoughts’ ([81] pg 9) troubling thoughts that an individual may be having, such as thinking they can never stop using substances or that everything is hopeless. Difficult situations and negative thoughts may result in troubling ‘emotions’ that an individual may be experiencing, such as feeling depressed or anxious [35]. The combination of negative thoughts and troubling emotions may then result in troubling ‘physical sensations’ an individual may be experiencing such as cravings and withdrawal, or somatic symptoms of anxiety such as muscle tension (e.g. [82]).

The result of negative thoughts, troubling emotions and physical sensations can then lead to ‘unhelpful behaviours’ that a person may engage in to alleviate the discomfort felt from their thoughts, emotions and physical sensations. In this instance, the unhelpful behaviours of most relevance are those related to the consumption of substances. However, other unhelpful behaviours may also be relevant, such as demonstrating aggressive behaviours, which may contribute to further difficult situations (e.g. [83, 84]). The additional domain of ‘lifestyle’ underpins all the other areas described above and includes issues such as employment, housing and social networks that directly affects our environment, thoughts, emotions, physical sensations and behaviours.

The LBM posits that when a substance dependent person encounters a difficult or challenging situation within their environment, they may be more likely to experience negative thoughts and associated distressing emotions such as stress, anxiety or low mood ([81] pg 9). These emotions can give rise to aversive physical sensations (including cravings for substances), that may in turn, motivate the individual to adopt unhelpful behaviors (including substance use) as a means of coping [85, 86]. Following their consumption of alcohol or drugs, or engagement in other dysfunctional coping behaviors, the individual may experience negative thoughts, such as guilt or shame [87, 88] which may in turn, trigger further negative emotions and attendant physical sensations, leading directly to the use of more substances and/or other coping behaviors [89, 90].

The LBM and the causal links between the domains contained within the model are currently being subjected to empirical testing via research into the effectiveness of the Breaking Free Online (BFO: [91–94]) and Breaking Free Pillars of Recovery (PoR: [95] treatment and recovery programs, the components of which are underpinned and structured by the LBM.

The BFO program provides online access to 22 technology-enhanced interactive evidence-based intervention strategies taken from evidence-based psychosocial interventions such as cognitive-behavioural therapy (e.g. [35, 96]) and mindfulness approaches (e.g. [97, 98]). Audio and visual technology is used to deliver intervention content that has traditionally been delivered via face to face interaction with a SUD practitioners or paper-based documents. The content of the program was developed through a review of the literature around evidence-based approaches for SUD, in conjunction with a consultation of those working within the SUD sector and also by those receiving treatment for SUD.

The PoR program is a 12-week intervention that has been developed specifically to support the needs of individuals with SUD and comorbid mild to moderate mental health difficulties such as anxiety, depression and panic disorder. The PoR program contains cognitive-behavioral interventions [35, 96], relapse prevention strategies (e.g. [11, 97]), in addition to community reinforcement approaches (e.g. [98, 99]) which aim to increase environmental reinforcement of abstinence and reduce environmental reinforcement of substance consumption. Mindfulness training [97] is also incorporated into the program which draw on the principle of helping the individual to exist more fully in the present moment instead of being concerned with the past or the future, and techniques drawn from acceptance and commitment therapy [100, 101].

Through the research being conducted into the effectiveness of the BFO and PoR and the feedback gained from both practitioners and individual’s receiving treatment for SUD, empirical data is being gathered to evidence the LBM and further develop it. It is hoped that the LBM may, in future, inform other behaviour change interventions that help individuals achieve a healthier, more functional and more balanced lifestyle that facilitates the acquisition of recovery capital and recovery progression.
